# De Novo Transcriptome Profiling of Brain Tissue from the Annual Killifish *Nothobranchius guentheri*

**DOI:** 10.3390/life11020137

**Published:** 2021-02-11

**Authors:** Zulfiia G. Guvatova, Maria S. Fedorova, Yulia S. Vershinina, Elena A. Pudova, Anastasiya V. Lipatova, Vsevolod V. Volodin, Natalya S. Gladysh, Artemiy T. Tokarev, Alexey B. Kornev, Vladislav S. Pavlov, Ildar R. Bakhtogarimov, Evgeny Y. Krysanov, Alexey A. Moskalev, George S. Krasnov, Anna V. Kudryavtseva

**Affiliations:** 1 Center for Precision Genome Editing and Genetic Technologies for Biomedicine Engelhardt Institute of Molecular Biology, Russian Academy of Sciences, 119991 Moscow, Russia; fedorowams@yandex.ru (M.S.F.); yulia_vershinina@list.ru (Y.S.V.); pudova_elena@inbox.ru (E.A.P.); lipatovaanv@gmail.com (A.V.L.); vsevolodvolodin@yandex.ru (V.V.V.); natalyagladish@gmail.com (N.S.G.); artemiy.tokarev@mail.ru (A.T.T.); vladislav1pavlov@gmail.com (V.S.P.); bakhtogarimov@gmail.com (I.R.B.); amoskalev@list.ru (A.A.M.); gskrasnov@mail.ru (G.S.K.); 2 Institute of Problems of Chemical Physics of Russian Academy of Sciences, 142432 Chernogolovka, Russia; abkornev@yandex.ru; 3 A.N. Severtsov Institute of Ecology and Evolution, Russian Academy of Sciences, 119991 Moscow, Russia; krysanov@sevin.ru

**Keywords:** *Nothobranchius guentheri*, aging model, transcriptome, mTOR, Torin 2, RNA-seq

## Abstract

*Nothobranchius* is a genus of small annual killifish found in Africa. Due to the relatively short lifespan, as well as easy breeding and care, *Nothobranchius* fish are becoming widely used as a vertebrate model system. Studying the genome and transcriptome of these fish is essential for advancing the field. In this study, we performed de novo transcriptome assembly of brain tissues from *Nothobranchius guentheri* using Trinity. Annotation of 104,271 potential genes (with transcripts longer than 500 bp) was carried out; for 24,967 genes (53,654 transcripts), in which at least one GO annotation was derived. We also analyzed the effect of a long-term food supplement with Torin 2, second-generation ATP-competitive inhibitor of mTOR, on the gene expression changes in brain tissue of adult *N. guentheri*. Overall, 1491 genes in females and 249 genes in males were differently expressed under Torin 2-supplemented diet. According to the Gene Set Enrichment Analysis (GSEA), the majority of identified genes were predominantly involved in the regulation of metabolic process, dendritic spine maintenance, circadian rhythms, retrotransposition, and immune response. Thus, we have provided the first transcriptome assembly and assessed the differential gene expression in response to exposure to Torin 2, which allow a better understanding of molecular changes in the brain tissues of adult fish in the mTOR pathway inhibition.

## 1. Introduction

Over the past decades, teleost fish due to experimental advantages in a laboratory setting has become a valuable vertebrate model for many fields of scientific research, including gerontology. Several age-related changes, including weakening immune system, increasing tumor risk, disruption of circadian rhythms, and alterations in locomotor activity, have been observed in widely known members of infraclass *Teleostei*, such as *Danio rerio* (zebrafish) and *Oryzias latipes* (medaka) [1,2,3]. However, despite the similarities between the biology of aging in fish and mammals, zebrafish and medaka have a strong regenerative ability and possible indeterminate growth [4]. In particular, the applicability of these species as a brain aging model has been questioned due to their ability to extensive adult neurogenesis and neuronal regeneration [5].

African annual fish of the genus *Nothobranchius*, so-called killifish, inhabit temporary pools filled during the monsoon season and are characterized by an extremely short life span. *Nothobranchius* fish is a novel promising model organism for aging research which reproduces many typical aspects of vertebrate brain aging, such as gliosis and decline of neurogenic activity in adults [6,7]. Another interesting feature of *killifish* in the context of comparative aging studies is the difference in the maximum life expectancy between species that could be associated with adaptation to different rain cycles in habitats [8]. *Nothobranchius furzeri* is the most investigated *Nothobranchius* species due to its shortest life span among the vertebrates. There are assembled genome and transcriptomes for several tissues of *N. furzeri*, including the brain [9]. Age-dependent changes in the brain transcriptome of *N. furzeri* have been recently described by Baumgart et al. [7]. On the other hand, not much is known about another *Nothobranchius* species, such as *N. guentheri*, which is considered the longest living species among the genus. The maximum laboratory life span described for *N. guentheri* was 2 years and 3 months [8], which is still much shorter compared to other popular vertebrate fish models, such as zebrafish with an average lifespan of 3 and a half years or medaka with a lifespan of around 4 years. One of the earliest fish models for aging research, *N. guentheri,* is responsive to pharmacological and lifestyle interventions and demonstrates changes in the expression of some age-related markers [10,11,12,13]. However, the changes taking place at the transcriptome level are not clearly understood.

In the present study, we performed *de novo* transcriptome assembly and analyzed the gene expression profile of brain tissue from adult *N. guentheri*. In addition, we were interested in studying the effect of a long-term food supplement with Torin 2 on the gene expression changes in the brain of *N. guentheri*. The mechanistic target of rapamycin (mTOR) is a highly evolutionarily preserved serine/threonine kinase. As a core component of two large functionally distinct multiprotein complexes termed mTORC1 (mTOR Complex 1) and mTORC2 (mTOR Complex 2), this kinase plays diverse roles in many cellular processes. Acting as a nutrient and energy sensor, mTORC1 promotes cell growth, ribosomal biogenesis, translation, and lipid synthesis in response to growth factors and nutrients. In addition, mTORC2 has been shown to regulate the cell survival and actin cytoskeleton organization [14]. In the brain, mTOR-regulated pathways are involved, among others, in specific processes, such as axonal sprouting, axonal regeneration, dendritic spine growth, and synaptic plasticity. Dysregulation of mTOR signaling is associated with the onset and progression of a broad range of brain disorders, including tumor, epilepsy, dementia, traumatic brain injury, and stroke [15,16]. Torin 2 is a highly selective, second generation ATP-competitive inhibitor of mTOR, that inhibits mTORC1-dependent T389 phosphorylation on serine/threonine kinase S6K. Unlike classical mTOR inhibitors, such as rapalogs, Torin 2 affects both mTORC1 and mTORC2 activity [17,18] and has a superior pharmacokinetic profile [19]. Our findings could contribute to better understanding transcriptomic changes associated with the inhibition of mTOR signaling in the adult brain using a novel vertebrate model, *N. guentheri.*

## 2. Materials and Methods

### 2.1. Fish Diet and Maintenance

The eggs of *N. guentheri* Zanzibar TAN 14-02 were obtained from a commercial supplier (Peter Covar, Brno, Czech Republic) and bred in the Center for Precision Genome Editing and Genetic Technologies for Biomedicine at the Engelhardt Institute of Molecular Biology. All *N. guentheri* experiments were carried out in accordance with the recommendations described in the Guide for the Care and Use of Laboratory Animals [20] and were approved by the Ethics Committee of the A.N. Severtsov Institute of Ecology and Evolution Russian Academy of Sciences (Experimental Research Regulatory Comission of Institute of Ecology and Evolution A.N. Severtsov, approval number 27, 9/10/2019). Torin 2 was synthesized and kindly provided by the Institute of Problems of Chemical Physics of Russian Academy of Sciences.

All the fish were bred under identical conditions after hatching. The fish were kept at 27 °C under 14:10 h day:night regime in a 1X E3 solution. To prepare an E3 stock solution (60X), 174 g NaCl, 8 g KCl, 29 g CaCl_2_, and 48.9 g MgCl_2_·6H_2_O were dissolved in 10 L distilled water. A week after hatching, the larvae were moved into the individual 1.5 L tanks. The feeding of fish larvae was carried out two times per day with newly hatched brine shrimp *Artemia salina*. At the age of sexual maturity (6 weeks), the fish were divided into experimental and control groups. The experimental group was switched to an experimental diet prepared as follows: An agarose gel (0.8%) containing *Artemia salina* nauplii and 1 mM Torin 2 was passed through a sieve with a mesh of approximately 1 mm^2^ for grinding. Each fish of the experimental group was fed twice a day with a freshly prepared experimental diet, so that the daily ration contained Torin 2 dose of 30 mg/kg of body weight. The uneaten agarose gel pieces were removed 30 min after addition to keep the water clean. Similarly, the control group was fed with shredded agarose gel pieces containing only *Artemia salina* nauplii.

### 2.2. RNA Isolation, Library Preparation, and Transcriptome Sequencing

Transcriptomic analysis was performed using *N. guentheri* at the age of 10 weeks. Thirty control (15 males and 15 females) and 33 experimental (18 males and 15 females) fish were prepared. Brains were dissected and stored at −80 °C until the RNA extraction. Experiments were performed at least in five replicates for each experimental variant. For convenience, the sample names were abbreviated (for example, NGF1E means *N. guentheri*, females, 1st replicate, experimental fish).

Total RNA was isolated from the pooled brain tissue samples (three brains per replicate) using the MagNA Pure Compact RNA Isolation Kit on a MagNA Pure Compact Instrument (Roche, Basel, Switzerland) according to the manufacturer’s protocol. The quantity of RNA was assessed using a Qubit^®^2.0 Fluorometer (Thermo Fisher Scientific, Waltham, MA, USA); quality control was carried out using a NanoDrop^®^ ND-1000 spectrophotometer (NanoDrop Technologies Inc., Wilmington, DE, USA) and Agilent 2100 Bioanalyzer (Agilent Technologies, Santa Clara, CA, USA). The A260/A280 ratios of RNA samples were 1.8–2.0. The RNA integrity number (RIN) of each sample was not less than 8.0.

For the cDNA library preparation, we used an Illumina TruSeq RNA Library Prep Kit v2 (low-throughput protocol) according to the manufacturer’s guidelines. The mRNA molecules containing poly(A) tails were isolated from 1 μg of total RNA by the poly-T oligo-attached magnetic beads, fragmented, and primed for cDNA synthesis. The first strand cDNA synthesis was performed using SuperScript^®^ II Reverse Transcriptase (Thermo Fisher Scientific, Waltham, MA, USA) and random primers. Then, cDNA was converted into the double-stranded (ds)cDNA and purified by AMPure XP beads. For the creation of blunt ends on the dscDNA, the end-repair reaction was performed. A single “A” nucleotide was added to the 3′ ends to avoid the ligation of blunt ends during the adapter ligation reaction. After the adapter ligation with RNA Adapter Indexes, supplied in the kit, the PCR (15 cycles) was performed to amplify the amount of DNA in the library. The concentration of the 21 obtained cDNA libraries was evaluated using a Qubit^®^2.0 Fluorometer. The quality was checked on an Agilent 2100 Bioanalyzer using a High Sensitivity DNA chip (Agilent Technologies). The cDNA libraries were normalized to 4 nM, pooled together in equal volumes, and sequenced with 75 bp single-end reads on the NextSeq 500 System (Illumina, San Diego, CA, USA). We obtained on average 25 million reads for each library. The sequencing data are available at the NCBI Sequence Read Archive (project ID PRJNA661435).

### 2.3. NGS Data Processing

The transcriptome assembly was performed with Trinity 2.9.0 [21] using Illumina reads derived from all 21 RNA-Seq libraries. The longest ORFs were predicted using TransDecoder 5.5.0. The annotation of assembled transcripts and ORFs was performed using the Trinotate 3.2.0 pipeline based on blastx/blastp mappings against UniProt and HMMER homology search against Pfam databases. From here, possible gene names, KEGG, and GO annotations were derived. The completeness of the transcriptome assembly was assessed with BUSCO 4.0.6 (in transcriptome mode) using four datasets: *Eukaryota*, *metazoa, vertebrata,* and *actinopterygii* (odb10).

Next, Illumina reads were mapped to the assembled transcripts using a bowtie2 [22] and quantified with RSEM [23]. The derived read counts per gene and per transcript were compared between the treatment and control groups with an edgeR Bioconductor package [24], separately for males and females, and pooled sex. The significance of differences observed between the two groups (control fish/Torin 2 treated fish) was assessed using a quasi-likelihood F-test and non-parametric Mann-Whitney U test (when the size of the groups allowed for this). Next, the Benjamini-Hochberg adjustment for multiple testing was applied to *p*-values to calculate the false discovery rate (FDR). The differences between groups were considered statistically significant if FDR was <0.05 and their magnitude was at least two-fold (|LogFC| ≥ 1). The GO enrichment analyses were performed using the goseq Bioconductor package. The KEGG pathway visualization was done using the pathview Bioconductor package.

## 3. Results

### 3.1. De Novo Transcriptome Assembly

A total of 536 million single-end Illumina reads (approximately 41 Gbases) were obtained from the 21 libraries derived from the samples of the male and female *N. guenteri* brain tissues. After trimming reads with trimmomatic, they were passed to the Trinity assembler. A total of 352,297 transcripts (i.e., contigs) corresponding to 288,989 potential genes (i.e., contig clusters) have been assembled. General assembly statistics are presented in Table 1.

**Figure 1:**
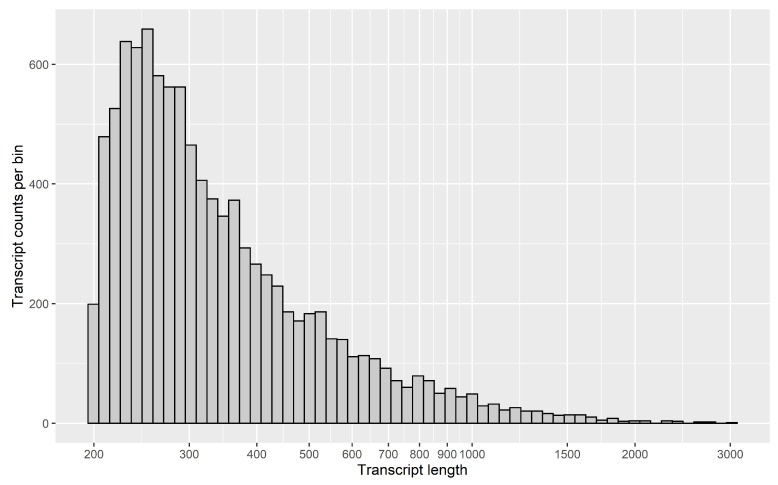
The histogram illustrating the distribution density of the assembled transcripts depending on their length (bp)**.**

The total number of genes with transcripts longer than 500 bp was 104,271. The largest assembled transcript size was 27,376 bp. The average GC content was approximately 47%. It is possible that such excess number of contigs (especially short) is due to the active transcription of intergenic spacers, intronic regions, including antisense or long non-coding RNAs. Possibly, they cannot be completely assembled even with such a high coverage (41 Gb), and are fragmented into several contigs. Indeed, from Figure 1 we see that most of the contigs are less than 500 bp (see also Table 1). Only 19% of contigs are >1000 bp and, therefore, can contain open reading frames (ORFs) encoding for proteins > 330 aa long. 

**Figure 2:**
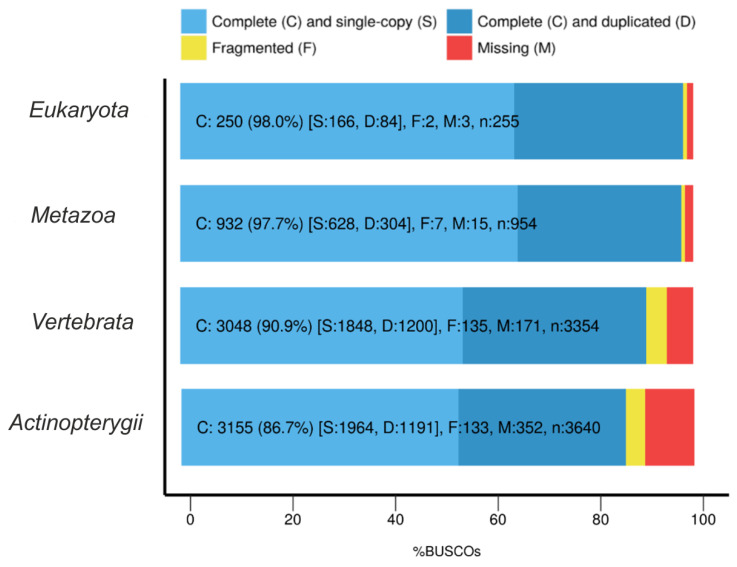
The results of the evaluation of transcriptome assembly completeness according to Benchmarking Universal Single-Copy Orthologs(BUSCO). Four datasets were used for the analysis: *Eukaryota, Metazoa, Vertebrata,* and *Actinopterygii*.

The completeness of the transcriptome assembly was evaluated by BUSCO [25]. The derived values were different when using different reference BUSCO datasets (Figure 2). For the high-level datasets (*Eukaryota* and *Metazoa*), the completeness was found at 98%, but for the *Vertebrata* and *Actinopterygii* (ray-finned fish) datasets, the completeness was significantly less—only 91 and 87%, respectively. This is quite expected and most possibly due to the fact that we only used one tissue for the transcriptome assembly. High-level BUSCO datasets (*Eukaryota* and *Metazoa*) mostly include more conservative and “basic” genes compared to the *Vertebrata* and *Actinopterygii* datasets. As a rule, these genes are more constitutively expressed regardless of the tissue type (for example, housekeeping genes are both highly conservative and highly expressed in various tissues).

Thus, single-end Illumina sequencing was sufficient to obtain a de novo transcriptome of brain *N.guentheri* with reasonable quality for the following analysis.

**Figure 3:**
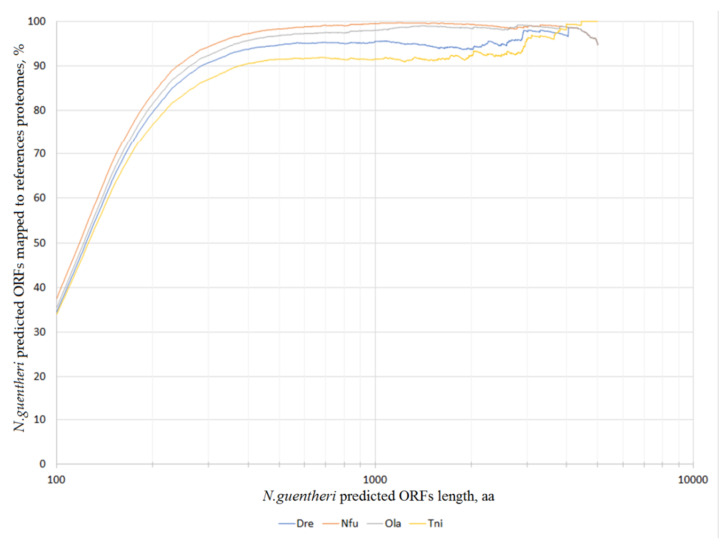
The percentage of predicted *N.guentheri* translated open reading frames (ORFs) successfully mapped to the reference proteomes of four organisms: *Danio rerio* (Dre), *Oryzias latipes* (Ola), *Tetraodon nigroviridis* (Tni), and *Nothobranchius furzeri* (Nfu) depending on the minimal predicted ORF length (aa). The longer the predicted protein, the more of these proteins are successfully mapped to the reference proteomes.

Next, using blastp, we mapped the predicted protein sequences to the proteomes of four different fish species (belonging to different orders), whose genomes are available in the NCBI database [26]: *Danio rerio* (zebrafish), *Oryzias latipes* (Japanese medaka), *Tetraodon nigroviridis* (spotted green pufferfish), and *Nothobranchius furzeri* (turquoise killifish). The mapping statistics (depending on a minimal encoded protein length) is shown in Figure 3. Without setting a threshold for the minimal protein length, only 30% of ORF translations have an homology in the reference proteomes. However, as the threshold increases to 250–300 aa, about 90–95% of the proteins find their blast hits. For the most closely related species, *N. furzeri*, the percentage of ORFs with protein homology reaches 99% (with a threshold length of about 700 aa). This is quite expected, since this organism belongs to the same genus as the subject of the present study. In second place is *O. latipes*, and in third place is *D. rerio*, despite the fact that the last one is a popular model organism with a well-annotated genome. *T. nigroviridis* ranks fourth, this may be linked to a lower quality of the genome assembly or annotation.

### 3.2. Transcriptome Annotation 

Using TransDecoder (http://transdecoder.github.io, last accessed on 10 February 2021), for 352,297 transcripts we found 163,185 ORFs with an average translated length of 271.1 aa, while the maximum length was 8786 aa. Of these, 115,024 proteins (70.5%) begin with a start codon and have a stop codon at the end (e.g., are completed), 26,294 proteins (16.1%) are under-assembled at the 5′-end of the transcript (do not have methionine at the beginning), 11,585 proteins (7.1%) do not have a stop codon (are most possibly under-assembled in the 3′-tail of the transcript), 10,281 proteins (6.3%) have neither one nor the other.

Among the 352,298 potential transcripts, which correspond to 288,989 potential genes, for 10,990 genes (5392 transcripts) there were at least one valid homology in the Pfam database [27], 10,151 genes (5672 transcripts) were successfully mapped against UniProt [28] with blastx [29], and 10,517 genes (5239 transcripts) were mapped with blastp [29]. 

**Figure 4:**
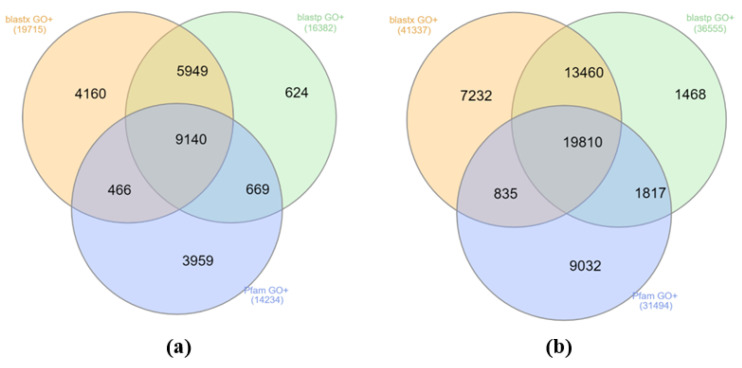
The number of genes (**a**) and transcripts (**b**) containing at least one gene ontology (GO) annotation derived based on either blastp/blastx mapping against UniProt and HMMER(profile hidden Markov model software for searching sequence databases for homologs) search against the Pfam-A database.

Additionally, 23,924 genes (52,566 transcripts) were mapped against the eggNOG database, 15,084 genes were assigned with the Kyoto Encyclopedia of Genes and Genomes (KEGG; including 3130 pathways from *D. rerio*, 18,884—KEGG Orthology pathways), 31,817 corresponding transcripts were assigned with KEGG (including 6712 pathways from *D. rerio*, 41,560—KEGG Orthology pathways). For 24,967 genes (53,654 transcripts), they were derived with at least one Gene Ontology (GO) annotation based on UniProt mapping or Pfam-A search (Figure 4). 

Figure 4 shows that all three search methods complement each other. Only for 36% the genes homology was detected by all three search methods at once. In general, blastx proved to be a more informative tool compared to blastp, since the search for blastx resulted in approximately 20% more homologies among UniProt compared to blastp. However, some of the homologies (2–3%) were detected only by blastp.

Moreover, 24,967 transcripts matched at least one GO term in the three major categories that were considered: Biological processes, molecular functions, and cellular components (Supplementary file 1). 

### 3.3. Gene Expression Changes Associated with the Torin 2 Treatment

**Figure 5:**
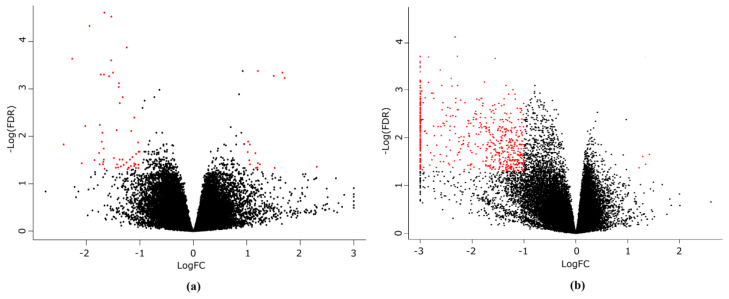
Volcano plots of differentially expressed genes (DEGs) between the controls and the Torin 2 treated *N. guentheri*. Volcano plots of DEGs in (**a**) males and (**b**) females. DEGs with two-fold or more differences in the expression level, that passed the FDR(false discovery rate) < 0.05 threshold are shown in red.

Next, we analyzed the effects of the Torin 2 treatment on the brain transcriptome of *N. guentheri*. Separate comparisons within males and females groups demonstrated that the changes in gene expression profiles caused by Torin 2 are much more pronounced in females than in males (Figure 5, Supplementary file 2). A total of 1491 transcripts (FDR < 0.05) showed differential expression in females in response to a diet with Torin 2 (363 of them had a BLAST hit in the UniProt database), while only 279 differentially expressed transcripts (108 of them had a BLAST hit in the UniProt database) were detected in males. 

Among the differentially expressed genes (DEGs) in males that passed the statistical significance (FDR < 0.05) and |LogFC| ≥ 1 thresholds, we detected a variety of genes involved in the regulation of the circadian rhythm such as *RORB, NFIL3, NR1D1, CIPC, DBP, BHE41,* and *TEF*. The Torin 2 treatment also altered the expression of several genes associated with the immune response (*DRA, HMR1,* and *HG2A*) and proteolytic processes (*CATS* and *CATK)*. The list of genes with a significantly changed expression in males is provided in Table 2.

In females, Torin 2 most influenced the expression of genes associated with mobile elements (*RTL1, PEG10, PO22, LITD1,* and *YTX1/2*). Most of these genes were downregulated under the Torin 2 diet. Moreover, specifically in females, the genes encoding for BED-type zinc finger domain-containing proteins (*ZBED1, ZBED4,* and *ZBED9*), which are mostly related to cell proliferation and originate from domesticated hAT DNA transposons and contribute to the regulation of various function in vertebrates, were downregulated in the Torin 2 treated group [30,31]. In addition, among the downregulated genes, we observed genes encoding the members of the protocadherin gene cluster (*PCDB1, PCDHGA, PCDHGB, PCDHD2,* and *PCDHGC*). The list of genes with significantly changed expression in females that passed the FDR < 0.05 threshold are provided in Table 3.

Predictably, many DEGs in response to the Torin 2 treatment were highly enriched in GO terms associated with the translation and cellular metabolism, including macromolecule, protein, and RNA metabolic processes. The gene set enrichment analysis also revealed alterations in “DNA binding” and “regulation of dendritic spine maintenance” GO terms. In males, we observed the downregulation of MHC protein complex genes, while in females, no statistically significant changes in this pathway were found. In females, several genes related to the regulation of muscle adaptation and multiple pathways associated with transposition were decreased. A complete list of enriched GO terms and direction of the changes in gene expression are presented in Supplementary file 2.

Surprisingly, we revealed that the top DEGs made up several hundred genes that do not have UniProt annotations and mostly have short transcripts <1000 bp. These genes were characterized by a high expression in control females, slightly lower—in control males, and very low (up to ten times lower)—in Torin 2 treated males and females. Compared to the annotated genes, they had a lower, but not much, “absolute” level of expression (average LogCPM was 4.3 for unannotated top DEGs and 5.4 for annotated ones).

## 4. Discussion

Neural tissues are characterized by extremely high metabolic requirements in comparison with other tissues [32,33]. The mTOR signaling, involved in controlling the balance between anabolic and catabolic processes, plays a critical role in maintaining the homeostasis of brain cells [15,34]. On the one hand, the mTOR activity is essential to neurogenesis, dendrite formation, synaptic formation, and plasticity [35]. Downregulation of mTOR signaling has been found in several models of the CNS neuron injury [15]. On the other hand, over-activation of mTOR complexes leads to a neurodevelopmental disorder, such as tuberous sclerosis (TS) and several neurodegenerative diseases, including Alzheimer’s, Parkinson’s, and Huntington’s diseases [36,37,38]. In these cases, the mTOR inhibition seems to be a reasonable strategy for treating brain pathologies. Previous studies of several models of neurodegenerative disorders have demonstrated that neuroprotective actions of mTOR inhibitors can be a result from the activation of autophagic processes and lysosomal biogenesis [39,40,41]. 

In the current work, we found that the Torin 2 treatment led to a slight increase in the expression of *ATG9A, ATG2A, ATG16, ATG4B, ATG12, SQSTM1,* and *ULK1* genes involved in autophagy and affected the activity of lysosomal proteases. A significant increase in the expression of cathepsins family genes (*CATS* and *CATK*) was also observed. Cathepsin K, encoded by the *CATK*, is a lysosomal cysteine protease which has been widely studied in the context of bone resorption [42]. Cathepsin K has been also shown to be present in the brain parenchyma and choroid plexus [43,44]. A deficiency in this cysteine protease leads to learning and memory deficits and can be associated with pathophysiology of schizophrenia and related neuropsychiatric disorders [44,45]. Cathepsin S encoded by the *CATS,* is a microglia-specific cysteine protease which is involved in the antigen presentation and is essential for the brain immune response [46]. Indeed, among the genes, in the expression which was altered in response to the Torin 2 treatment, we identified that *HG2A, HMR1,* and *DRA* were related to the major histocompatibility complex class II (MHC II). Moreover, the pathway enrichment analysis revealed the modulation of antigen processing and presentation, and NF-kappa B signaling pathway. Although the brain is regarded as an immuno-privileged site due to the muted inflammatory response, there is an accumulated evidence that immune signaling at the brain barriers may be contributing normal as well as pathological processes [47]. It was previously shown that the treatment with the mTOR inhibitor rapamycin not only promotes CD8 memory T cells development, but also improves the quality of memory CD8 T cells [48,49,50]. Our findings are consistent with Xu et al., who showed that this effect may be mediated by an increased autophagy activity due to the inhibition of the mTOR pathway [51]. However, in the present work, we observed that the effect of Torin 2 was strongly gender-specific.

Surprisingly, we found that the Torin 2 treatment led to a decrease in the expression of many genes involved in the transposition of transposable elements (TEs). Accumulating evidence suggests that TEs can be transcribed and mobilized both in the developing brain and during adult neurogenesis [52,53]. Aberrant TE activation, in particular, activation of LINE-1, has been hypothesized to be a cause of somatic mosaicism in the brain and can be involved in the pathogenesis of both neurodevelopmental and neurodegenerative disorders, such as cerebellar ataxia, Rett syndrome, schizophrenia, and depression [54,55]. 

Our results also imply the role of Torin 2 in the regulation of dendritic spine maintenance. Dendritic spines are the structures critical for synaptic plasticity and behavior [56]. The members of the γ-Protocadherin (Pcdh) gene cluster involved in dendritic spine growth and neuronal dynamics were downregulated. Protocadherins are essential for the function of specific cell-cell neural connections and contribute to the survival of multiple neuronal cell types [57,58,59]. According to recent research, the loss of Pcdhs in cortical neurons does not affect their survival or result in reduced synaptic density, but severely reduces dendritic arborization [60].

## 5. Conclusions

In conclusion, the present study provides the first brain transcriptomic view of adult fish *N. guentheri*. In addition, owing to the fundamental role of mTOR in the functioning of brain cells, we assessed the transcriptomic profile in the brain tissue of fish *N. guentheri* under modulation of the mTOR pathway using the mTOR inhibitor Torin 2. Our results show that a long-term food supplement with Torin 2 leads to changes in the expression of many genes involved in the regulation of dendritic spine maintenance, various metabolic processes, circadian rhythms, transposition, autophagy, and immune response. However, the effect of Torin 2 was strongly gender-specific. Thus, the results of the study allow a better understanding of the possible molecular mechanisms in vertebrate brains. Moreover, considering species-specific differences in the genus of *Nothobrancius*, obtained RNA-Seq data can be further used in comparative aging research.

## Figures and Tables

**Table 1:** Overall assembly statistics of *N. guenteri* transcriptome.

Feature	Value
total length, bp	285,906,387
total length, bp (only transcripts > 500 bp)	217,410,128
genes (any length)	288,989
genes (transcripts > 500 bp)	104,271
transcripts (any length)	352,297
transcripts > 500 bp	127,262
transcripts > 1000 bp	66,471
transcripts > 5000 bp	6322
transcripts > 10,000 bp	604
transcripts > 25,000 bp	5
largest transcript, bp	27,376
N50, bp	2539
N75, bp	1231
L50, bp	24,581
L75, bp	55,134
GC, %	47.07

**Table 2:** Genes with significantly changed expression in the Torin 2 treated male fish *N. guentheri*. The embedded heatmap (blue-red gradient) show the relative gene expression profiles (log-scaled ratio of the expression level in a current sample to the average level across all the samples). Blue—expression level is below the average, red—above the average. LogFC—average binary logarithm of the expression level ratio between the treated and control organisms; LogCPM—binary logarithm of average read counts per million (CPM); FDR value—adjusted *p*-value for multiple testing.

Gene ID	Top BLAST Hit in UniProt	Gene Name	Control					Torin 2						LogFC	LogCPM	FDR
*TRINITY_DN217860_c0_g1*	*CATK*	Cathepsin K												1.05	4.1	0.032
*TRINITY_DN6195_c0_g1*	*CATS*	Cathepsin S												1.20	4.8	0.046
*TRINITY_DN369_c0_g1*	*HMR1*	Major histocompatibility complex class I-related gene protein												1.16	6.6	0.023
*TRINITY_DN118978_c0_g1*	*HG2A*	HLA class II histocompatibility antigen gamma chain												1.06	4.6	0.039
*TRINITY_DN217842_c0_g1*	*DRA*	Mamu class II histocompatibility antigen, DR alpha chain												1.02	5.1	0.021
*TRINITY_DN6030_c0_g1*	*CDN1B*	Cyclin-dependent kinase inhibitor 1B												1.21	4.9	0.037
*TRINITY_DN3092_c0_g1*	*HEBP2*	Heme-binding protein 2												−1.03	5.1	0.013
*TRINITY_DN17955_c0_g1*	*PROD*	Proline dehydrogenase 1												−0.85	4.3	0.046
*TRINITY_DN121258_c0_g1*	*PROF1*	Profilin-1												1.25	5.3	0.039
*TRINITY_DN10729_c0_g1*	*SMU1*	WD40 repeat-containing protein SMU1												−1.57	3.8	0.001
*TRINITY_DN151812_c0_g1*	*TEF*	Transcription factor VBP												−1.53	4.8	0.000
*TRINITY_DN918_c0_g1*	*BHE41*	Class E basic helix-loop-helix protein 41												−1.50	5.2	0.000
*TRINITY_DN217450_c0_g1*	*CIART*	Circadian-associated transcriptional repressor												−1.54	3.2	0.000
*TRINITY_DN6901_c0_g1*	*CIPC*	CLOCK-interacting pacemaker												−1.11	3.2	0.038
*TRINITY_DN13536_c0_g1*	*DBP*	D site-binding protein												−1.25	4.8	0.000
*TRINITY_DN3883_c0_g1*	*NFIL3*	Nuclear factor interleukin-3-regulated protein												1.71	4.4	0.001
*TRINITY_DN221983_c0_g1*	*NR1D1*	Nuclear receptor subfamily 1 group D member 1												−1.67	4.0	0.000
*TRINITY_DN2234_c0_g1*	*NR1D2*	Nuclear receptor subfamily 1 group D member 2												−0.91	5.9	0.002
*TRINITY_DN13399_c0_g1*	*PER1*	Period circadian protein homolog 1												−1.39	4.2	0.001
*TRINITY_DN12790_c0_g1*	*PER2*	Period circadian protein homolog 2												−1.32	4.5	0.001
*TRINITY_DN2073_c0_g1*	*RORB*	Nuclear receptor ROR-beta												1.21	5.5	0.000

**Table 3:** Genes with significantly changed expression in the Torin 2 treated female fish *N. guentheri*. For details, see the legend for Table 2.

Gene ID	Top BLAST Hit in UniProt	Gene Name	Control					Torin 2					LogFC	LogCPM	FDRvalue
*TRINITY_DN9769_c0_g1*	*PCDB1*	Protocadherin beta 1											−1.01	3.9	0.003
*TRINITY_DN30433_c0_g1*	*PCDHGB*	Protocadherin gamma subfamily B											−0.92	2.9	0.002
*TRINITY_DN29438_c0_g1*	*PCDHGA*	Protocadherin gamma subfamily A											−1.85	1.8	0.005
*TRINITY_DN221187_c0_g1*	*PCDHD2*	Protocadherin delta 2											−1.79	2.2	0.002
*TRINITY_DN10099_c0_g1*	*PCDHGC*	Protocadherin gamma subfamily C											−1.43	3.1	0.002
*TRINITY_DN5709_c0_g3*	*ZBED1*	Zinc finger BED domain-containing protein 1											−1.21	4.4	0.0010
*TRINITY_DN32433_c0_g1*	*ZBED4*	Zinc finger BED domain-containing protein 4											−1.53	1.6	0.01
*TRINITY_DN11566_c0_g2*	*ZBED9*	SCAN domain-containing protein 3											−1.22	4.8	0.01
*TRINITY_DN28479_c0_g1*	*LITD1*	LINE-1 type transposase domain-containing protein 1											−2.22	1.5	0.002
*TRINITY_DN5063_c0_g1*	*PEG10*	Retrotransposon-derived protein PEG10											−1.37	4.0	0.009
*TRINITY_DN6282_c0_g1*	*RTL1*	Retrotransposon-like protein 1											−1.45	4.2	0.007
*TRINITY_DN4053_c0_g1*	*YTX1*	Transposon TX1 uncharacterized 149 kDa protein											−1.37	3.7	0.003
*TRINITY_DN18778_c0_g1*	*PO22*	Retrovirus-related Pol polyprotein from type-1 retrotransposable element R2											−0.91	2.8	0.05
*TRINITY_DN20585_c0_g1*	*FMRF*	FMRF-amide neuropeptides											−2.88	4.5	0.008
*TRINITY_DN28618_c0_g1*	*NEFH*	Neurofilament heavy polypeptide											−1.05	2.8	0.002
*TRINITY_DN36218_c0_g1*	*CSMD1*	CUB and sushi domain-containing protein 1											−1.83	2.2	0.006
*TRINITY_DN20186_c0_g1*	*DYH10*	Dynein heavy chain 10, axonemal											−1.32	2.4	0.004
*TRINITY_DN23456_c0_g1*	*FR1L6*	Fer-1-like protein 6											1.35	2.2	0.04
*TRINITY_DN20670_c1_g1*	*NMDE2*	Glutamate receptor ionotropic, NMDA 2B											−1.18	2.5	0.01
*TRINITY_DN19802_c0_g1*	*SRFBP1*	Serum response factor-binding protein 1											−3.95	2.5	0.01

## Data Availability

NCBI sequence read archive (project ID PRJNA661435).

## Supplementary Materials

The following are available online at https://www.mdpi.com/2075-1729/11/2/137/s1. Supplementary file 1: GO classification of transcripts (genes) from *N. guentheri* brain tissue; Supplementary file 2: The list of enriched pathways (*p*-value for gene ontology GSEA < 0.05) of differentially expressed genes associated with the Torin 2 treatment in females and males of *Nothobranchius guentheri*.
